# A dynamic model for the outbreaks of hand, foot, and mouth disease in Taiwan

**DOI:** 10.1017/S0950268815002630

**Published:** 2015-11-16

**Authors:** C.-C. LAI, D.-S. JIANG, H.-M. WU, H.-H. CHEN

**Affiliations:** 1 Emergency Department, Taipei City Hospital, Ren-Ai Branch, Taiwan; 2 Graduate Institute of Epidemiology and Preventive Medicine, College of Public Health, National Taiwan University, Division Biostatistics, Taipei Taiwan; 3 Field Epidemiology Training Program, Centres for Disease Control, Taiwan

**Keywords:** Compartmental model, enterovirus 71, hand, foot, and mouth disease (HFMD), reproductive number, SIR model

## Abstract

The first large outbreak of hand, foot, and mouth disease (HFMD) with severe complications primarily caused by enterovirus 71 was reported in Taiwan in 1998. Surveillance of HFMD to evaluate the spread of HFMD with and without infection control policy is needed. We developed a new dynamic epidemic Susceptible-Infected-Recovered (SIR) model to fit the surveillance data on containing valuable information on the severity of HFMD in order to accurately estimate the basic reproductive number (*R*_0_) of HFMD. After fitting the empirical data, in conjunction with other relevant parameters extracted from the literature, the estimated transmission coefficients were close to 5 × 10^−7^ (per day) and the proportion of severe HFMD cases ranged between 0 and 0·0036 (per day). Taking into account the distribution of all parameters considered in our dynamic epidemic model, the *R*_0_ computed was 1·37 (95% confidence interval 0·24–5·84), suggesting a higher likelihood of the spread of HFMD if no infection control policy is provided. The isolation strategy against the spread of HFMD not only delayed the epidemic peak with the delayed time ranging from 4 weeks for only 20% isolation to 47 weeks for 100% isolation but also reduced total number of HFMD cases with the percentage of reduction ranging from 1·3% for only 20% isolation to 13·3% for 100% isolation. The proposed model can also be flexible for evaluating the effectiveness of two other possible policies for containing HFMD, quarantine and vaccination (if the vaccine can be developed).

## INTRODUCTION

Hand, foot, and mouth disease (HFMD) is, to a large extent, caused by coxsackievirus A16 (CVA16) and, to a lesser extent, associated with other serotypes such as A4, A5, A9, A10, B2, and B5 viruses as well as echovirus and enterovirus 71 (EV71) [1–3]. Humans are the only natural host for these enteroviruses that are transmitted through two possible modes, personal oral–faecal route and oral–oral route [4]. The majority of diseases afflicting children are typically benign and self-limiting but HFMD caused by EV71 may be more likely to result in severe complications such as acute flaccid paralysis, encephalitis, meningitis, myocarditis and pulmonary oedema.

Large outbreaks associated with EV71, first described in 1974 [5], have been heralded in Japan since the early 1970s [6]. After around two decades, EV71 epidemic activity has been spread throughout the Asia-Pacific region producing large outbreaks such as the 1998 Taiwanese HFMD outbreak caused by two main types of virus, CVA16 and EV71. The age distribution and clinical features of infections (such as the proportion of HFMD and herpangina) between EV71 and CVA16 were similar. It is therefore difficult to clinically distinguish between HFMD caused by EV71 and that caused by CVA16, not to mention the asymptomatic but still infectious HFMD cases. In a Taiwanese seroepidemiological study of 46% people of all ages who were infected with EV71, about 71% showed no clinical symptoms [7]. The contrast between both types is that illnesses resulting from EV71 were more likely to lead to a significantly greater frequency of serious complications and fatalities than those caused by CVA16 [8, 9]. It is therefore important to make use of HFMD with severe complications to back-estimate symptomatic and asymptomatic but infectious cases resulting from infectives in the absence of intervention in order to calculate the basic reproductive number (*R*_0_, the expected number of secondary cases produced by an index case in a completely susceptible population).

*R*_0_ is a useful indicator for assessing the spread of HFMD because it is a function of the number of contacts per unit time, the probability of transmission per contact, and the duration of the infectious period [10]. If *R*_0_ >1, an outbreak occurs. However, if *R*_0_ <1, an outbreak is unlikely. Thus, if a sustained disease control reduces the transmission intensity by a factor that exceeds *R*_0_, HFMD will eventually be eliminated. Although *R*_0_ is widely estimated via the Susceptible-Infected-Recovered (SIR) model for other infectious diseases [10], its application in delineating the natural course of the infection–disease process and evaluating the preventive strategy containing HFMD is still limited because it is difficult to distinguish the pathogenic HFMD cases (including asymptomatic but still infectious cases and symptomatic cases) attributed to different causes of serotypes as indicated above from the empirical surveillance data. Therefore, it is intractable to estimate the relevant parameters for deriving *R*_0_ using this traditional compartmental model. To cope with this problem, we developed a new modified SIR model by adding information on clinical symptomatic and severe cases to fit the empirical surveillance data in order to accurately estimate the parameters governing the evolution from susceptibility to infection, recovery, and finally the development of severe HFMD. These parameters, while providing information on baseline infection and disease process related to the spread of HFMD in the absence of intervention, enables assessment of the effectiveness of infection control policy such as isolation, quarantine, and even vaccination if the vaccine can be appropriately deployed in containing the outbreak of HFMD. The aims of this article consist of two parts. In the development and estimation process, we first buid up the new proposed dynamic model. We then made use of empirical data from a wave of outbreaks on HFMD to train the parameters from susceptible, infection, asymptomatic disease, symptomatic disease until the final sequelae, recovery, HFMD, herpangina, and severe HFMD. We were able to project the value of the basic *R*_0_ without effective infection control strategy based on parameters estimated above. In the application part, the impact of HFMD (e.g. the delayed time for the spread of HFMD and a decrease in the number of HFMD cases) can be evaluated by the different policies of the isolation strategy.

## MATERIALS AND METHODS

### Data sources

In Taiwan, the government has established a physician-based sentinel surveillance system, representing 8·7% of primary physicians for HFMD since 1998; all severe cases of enteroviruses must be reported to the Centers of Disease Control (CDC) through a hospital-based reporting system [9, 11]. A severe case was defined as follows: patients were hospitalized if, in addition to HFMD or herpangina, they showed signs or symptoms indicating more serious illness including high fever (body temperature ⩾38 °C), vomiting, tachypnoea, and indications of neurological complications or cardiopulmonary complications [11]. Active household investigations of secondary clinical cases were performed after each severe case was reported. There was no change over time regarding the surveillance system.

**Fig. 1.**
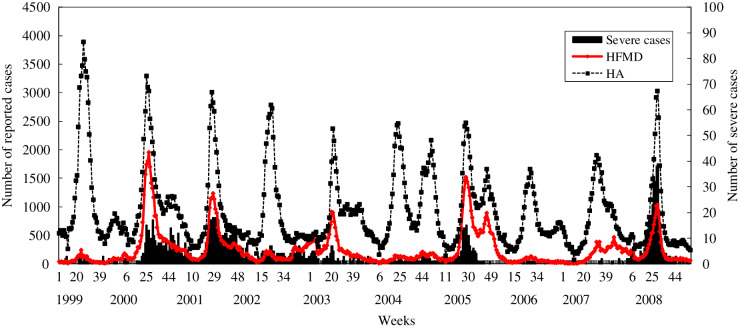
The reported cases of hand, foot, and mouth disease (HFMD) or herpangina (HA) in a physician-based sentinel surveillance system and the severe cases of HFMD or HA in Taiwan from 1999 to 2008.

The data of HFMD obtained from the Taiwan CDC were used to estimate the parameters pertaining to the compartmental SIR model (see Supplementary material). Figure 1 shows the number of patients with HFMD or herpangina reported by the sentinel surveillance system between 1999 and 2008. We modelled the results of previous outbreaks of HFMD to estimate the basic reproductive number (*R*_0_) in 2000, 2001, 2005 and 2008 due to the large outbreaks of severe cases with HFMD in these calendar years.

### Model specification

**Fig. 2.**
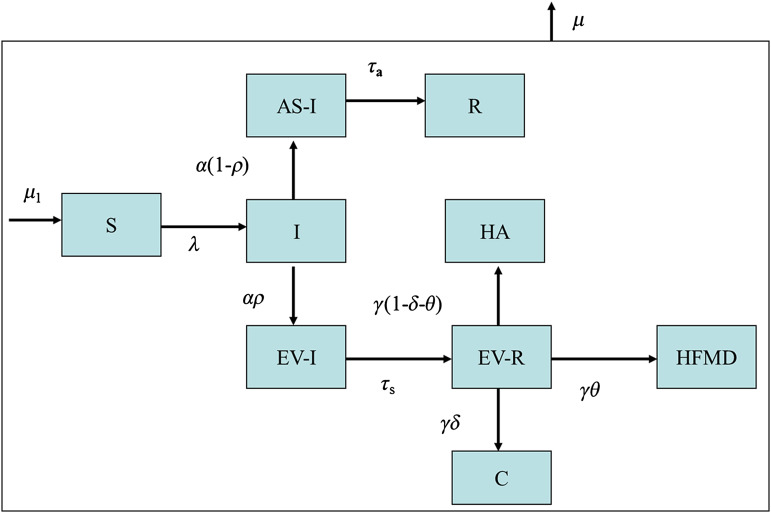
HFMD model. S, Susceptible; I, infectious cases before developing symptoms; AS-I, asymptomatic cases with infectiousness; R, asymptomatic subjects with immunity after infection; EV-I, infectious cases after developing symptoms; EV-R, symptomatic cases with immunity; HA, cases with herpangina or other symptoms; HFMD, cases with hand, foot, and mouth disease; C, severe cases due to HFMD virus; *μ*_1_, birth rate; *μ*, death rate; *λ*, force of infection; *α*, transfer rate from I to EV-I or AS-I; *ρ*, proportion of symptomatic cases; *τ*_a_, rate of recovery from AS-I; *τ*_s_, recovery rate from EV-I; *γ*, transition rate from EV-R; *θ*, ratio of the HFMD; *δ*, proportion of severe cases to all symptomatic cases with the enterovirus which result in HFMD.

We modified the SIR model to evaluate the behaviour of the virus causing HFMD and the severe cases obtained from the surveillance system of Taiwan CDC. The compartmental model based on the characteristics of HFMD is shown in Figure 2 with the detailed relationships and notations. In brief, the population was divided into nine compartments namely, individuals susceptible to enterovirus (S), infectious cases before developing the disease (I), asymptomatic disease with infectiousness (AS-I), immunity after infected AS-I (R), infectious cases after developing symptoms (EV-I), symptomatic cases with immunity (EV-R) after recovery, typical cases with HFMD, severe cases and other mild symptoms or herpangina. Figure 2 shows the rates of transitions between these nine states. The model begins with the state of susceptible individuals (S) and then enters the infectious state (I) when infected. The subsequent transitions include I to AS-I or EV-I. Note that AS-I may end up with R. The most novel of this compartmental model is to add the EV-R compartment that represents a transient state for the recovery from EV-I to EV-R. If there is no recovery, the final outcome would be ascertained as herpangina, HFMD, or severe cases. Note that the exact dwelling time remaining in the infectious period in the EV-I state would be difficult to estimate if the EV-R compartment is lacking. From the viewpoint of population, the number of susceptible individuals is also affected by the death rate of the population. In addition, HFMD is a common infectious disease of infants and children, which could be largely affected by the birth rate.

The nonlinear ordinary differential equations of the HFMD model were used to describe the dynamic changes of each state. The expressions are detailed in the Supplementary material. The model assumes that the durations of these compartmental periods are exponentially distributed, giving the average duration by taking the reciprocal of the transition rates of leaving these states. We assume all enteroviruses causing HFMD lead to the similar distributions of clinical symptoms except for severe cases.

### Derivation of basic reproductive number (*R*_0_)

We used a next-generation method [10] to derive *R*_0_ in models with several disjointed compartments. The detailed process of deriving *R*_0_ is elaborated upon in the second part of the Supplementary material [see equations (10)–(12)]. The *R*_0_ derived from our model is a function of the transmission rate (*β*), the death rate (*μ*), the transition rate (*α*) from infectious cases before developing symptoms (I) to infectious cases after developing symptoms (EV-I) or asymptomatic cases with infectiousness (AS-I), the proportion of symptomatic cases (*ρ*), the rate of recovery from AS-I (*τ*_a_), and the rate of recovery from EV-I (*τ*_s_).

The formula of deriving *R*_0_ is summarized as follows:

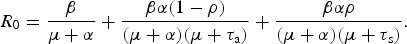


The numerators of the formula consist of three parts, the transmission rate before developing disease (*β*), the product of the transmission rate with the rate of developing asymptomatic disease [*β* × *α* × (1 – *ρ*)], and the product of the transmission rate with the rate of developing symptomatic disease (*β* × *α* × *ρ*). The denominator of the first part is the inverse of the death rate plus the transition rate from infectious cases before developing symptoms (*μ* + *α*). In addition to *μ* + *α*, the denominators of the second and third parts are incorporated with the inverse of the death rate plus the rates of recovery for asymptomatic cases (*μ* + *τ*_a_) and the inverse of the death rate plus the rates of recovery for symptomatic cases (*μ* + *τ*_s_), respectively. We estimated the parameters of this dynamic model proposed in Figure 2 using Matlab version 7.10·0·499 (MathWorks, USA).

### Input of model parameters

Table 1 shows the input of parameters used in the modified dynamic SIR model shown in Figure 2. The population size (N) was identical to the Taiwanese population. The birth and death rates were obtained from Taiwan public health vital statistics [12]. The proportion of symptomatic cases was ~94% in children and 47% in adults [13]. EV71 seroprevalence rates ranged from 57% to 67% in adults and children aged >6 years, from 0% to 36% in children aged <3 years and from 26% to 51% in children aged 3–6 years [7]. Therefore, the proportion of susceptible (S) individuals in the total population was estimated to be ~45%.

**Table 1.** The parameters of model for HFMD outbreaks in Taiwan

Variable	Range	Outbreak 2000	Outbreak 2001	Outbreak 2005	Outbreak 2008	Outbreak 2008*
Parameter setting						
* N*	—	22 092 387	22 276 672	22 689 112	22 958 360	22 958 360
* μ*_1_	—	3·8 × 10^−5^	3·2 × 10^−5^	2·5 × 10^−5^	2·37 × 10^−5^	2·37 × 10^−5^
* μ*	—	1·6 × 10^−5^	1·6 × 10^−5^	1·7 × 10^−5^	1·71 × 10^−5^	1·71 × 10^−5^
S	—	45%	45%	45%	45%	45%
E	—	1·4%	1·4%	1·4%	1·4%	1·4%
* α*	>0·167	0·35	0·35	0·35	0·35	*γ*(32·92, 0·01)
* τ*_a_	0·028–0·125	0·08	0·08	0·08	0·08	*γ*(28·44, 0·0028125)
* τ*_s_	0·028–0·125	0·08	0·08	0·08	0·08	*γ*(28·44, 0·0028125)
* γ*	—	1	1	1	1	1
* ρ*	0·47–0·94	0·7	0·7	0·7	0·7	*β*(0·6225, 0·26048)
* θ*	0·45–0·49	0·47	0·47	0·47	0·47	0·47
Infected number at beginning of outbreak	—	80	240	110	500	500

*N*, Number of total population; *μ*_1_, birth rate; *μ*, death rate; S, proportion of susceptible; E, proportion of exposed to infectious subjects; *α*, transition rate from *I* to EV-I or AS-I; *τ*_a_, rate of recovery from AS-I; *τ*_s_, recovery rate from EV-I; *γ*, transition rate from EV; *ρ*, proportion of symptomatic cases; *θ*, ratio of HFMD; *δ*, proportion of severe cases; *β*, transmission coefficient.

* The parameters for calculating *R*_0_ with Markov Chain Monte Carlo.

As we assumed that most of the other parameters except the transmission coefficient (*β*) and the proportion of severe HFMD cases (*δ*) were stable across studies, they were extracted from the literature. The parameters on the transition rate from I to EV-I or AS-I (*α*), the recovery rate from AS-I (*τ*_a_), and the recovery rate from EV-I (*τ*_s_), were elicited using the following information. The incubation period of HFMD is about 1–7 days [14]. The infectious period typically occurs during the acute stage of HFMD, and it persists for several weeks [15]. If the infectious period of HFMD is the same as that of polio (14–42 days) [15], we estimated the asymptomatic period with infectiousness to be about 0–6 days, and the symptomatic period with infectiousness to be about 8–36 days. The average time in the infectious period (I) before the symptomatic phase was suggested to be 2·86 days (1/[*αρ* + *α*(1 – *ρ*)]), and the average time in the symptomatic phase with infectiousness (EV-I) was assumed as 12·5 days (1/*τ*_s_). It was inferred that the range of *α* (the transition rate from I to EV-I or AS-I) was at least 0·167 (per day), and the best-case estimate was therefore assigned as 0·35. The range of *τ*_a_ and *τ*_s_ was between 0·028 and 0·125, and the best-case estimate was therefore assigned as 0·08. The best-case estimate of the proportion of symptomatic cases (*ρ*) was assigned as 0·7 [6, 7]. As mentioned before, illnesses resulting from EV71 were more likely to be severe than those resulting from CVA16, but the distributions of HFMD, herpangina or other mild symptoms were similar [8]. This presupposes that the rate of transition from EV-R to herpangina was similar to that of HFMD but different from that of EV-R to severe cases (see Fig. 2).

Although it was assumed that these parameters cited in the literature were stable across studies, they would be conjoined with the two parameters (such as transmission coefficients and the proportion of severe HMFD cases) to examine whether they were fitted well with the empirical data. In almost all of the cases, the children were aged <6 years, and ~75% of the children were aged <3 years [8, 13]. CVA16 or EV71 resulted in 45–49% HFMD, 10% herpangina, and 6·3% (CVA16) in ~21% (EV71) of severe cases [8, 13, 16]. Such information gives the range of the proportion of severe HFMD cases (*δ*).

### Statistical analysis

Given the number of the total population and the birth and death rates each year in Taiwan, we applied the proposed dynamic epidemic model to fit surveillance to data of HFMD to train the parameters of transmission coefficients and the proportion of severe HFMD cases (*β, δ*) in conjunction with the other parameters indicated above.

We then validated the results of applying the parameters trained from the surveillance data to assess whether the number of observed severe HFMD cases was close to that of the predicted severe cases with the Pearson's *χ*^2^ test. If the observed value was <5, we combined the adjacent values until it was >5.

As the parameters used in the model-fitting process were assigned in a deterministic manner, we attempted to take into account the uncertainty of these parameters while basic reproductive numbers (*R*_0_) were computed. We therefore considered using the Bayesian Markov Chain Monte Carlo (MCMC) method to assign the major parameters (*α, τ*_a,_ *τ*_s,_ *ρ, β*) with the proper statistical distributions to derive the posterior distribution of *R*_0_ based on the empirical data from the outbreak in 2008 (Table 1; see also Supplementary material). We assigned *β* to an inverse gamma distribution, *α* to the gamma distribution, *τ*_a_ and *τ*_s_ to gamma distributions and *ρ* to beta distributions. All of the computation procedures were implemented using WinBugs version 1.4.3 (www.mrc-bsu.cam.ac.uk/software/bugs/).

### Impact of isolation strategy against HMFD

HFMD cannot be prevented by immunization because vaccine against these viruses has not been developed yet. Therefore, information about how to properly wash hands, early diagnosis and treatment of HFMD, and suspension of school classes during the endemic period was used to stop the spread of HFMD in Taiwan.

It was easy to evaluate the effects of the intervention, such as isolation (suspension of class), on HFMD disease prevalence by adding the isolated factor denoted by Ω, indicating the isolation rate of symptomatic HFMD in the model. Therefore, *λ*(*t*) in the dynamic model was changed to the following equation:

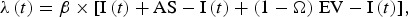

where I(*t*) denotes the number of infectious cases before developing the disease at time *t*, which denotes the numbers of asymptomatic disease with infectiousness (AS-I) at time *t*; EV-I(*t*) denotes the number of infectious cases after developing symptoms at time *t*, and Ω denotes the proportion of isolated EV-I. We will demonstrate how the five isolation strategies with respective proportions, 0·2, 0·5, 0·7, 0·9 or 1, delayed the outbreak of HFMD and also reduced the disease burden (i.e. total number of HFMD cases) in the epidemic curves of coming years. All these simulations were conducted using Matlab version 7.10.0.499 (MathWorks).

## RESULTS

The parameters estimated by fitting the proposed HFMD dynamic model to the empirical data are listed in Table 2. The transmission coefficient (*β*) (per day) was 5·7 × 10^−7^ in the 2000 outbreak, 5·6 × 10^−7^ in the 2001 outbreak, 4·5 × 10^−7^ before 34 weeks, 1·2 × 10^−6^ after 34 weeks in the 2005 outbreak, and 5·3 × 10^−7^ before 25 weeks, 2 × 10^−7^ between 26 and 33 weeks and 4 × 10^−7^ after 34 weeks in the 2008 outbreak. The estimated proportion of severe HFMD cases ranged between 0 and 0·0036 (per day). We also assumed the asymptomatic cases with infectiousness (AS-I) were equal to those of the symptomatic phase with infectiousness (EV-I). Therefore, the total time spent in I and EV-I was ~15·36 days (1/*α* + 1/*τ*_s_). The simulated and reported HFMD cases are shown in Figure 3, which indicated a good fit with visual inspection. Figure 4 shows the results of applying the parameters to validate whether the predicted severe HFMD cases were close to those observed. The goodness-of-fit test resulted in a *χ*^2^ = 5·84 with 8 degrees of freedom (*P* = 0·67) for severe cases in 2005, and a *χ*^2^ = 31·83 with 25 degrees of freedom (*P* = 0·16) for severe cases in 2008 (Supplementary material). These tested results indicated no evidence of absence of goodness of fit. Note that the simulated values were not in good agreement with the observed good matches in 2000 and 2001 (*P* < 0·0001) because the initial sentinel surveillance system for HFMD was unstable or different aetiological viruses existed.

**Fig. 3 (a, b).**
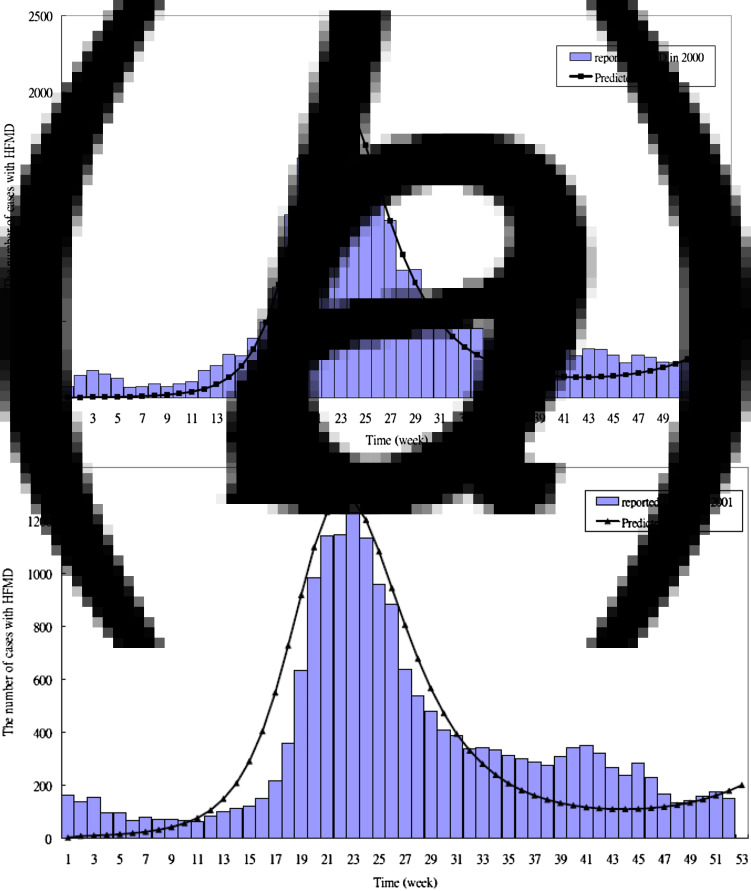
The observed and predicted HFMD cases in Taiwan in (*a*) 2000; (*b*) 2001.

**Fig. 3 (c, d).**
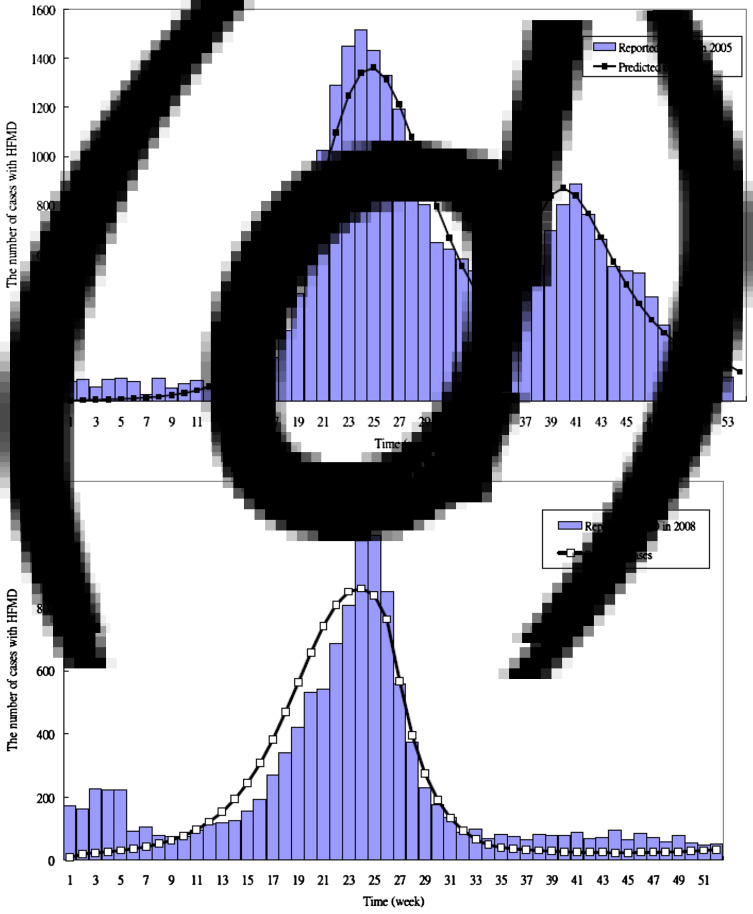
The observed and predicted HFMD cases in Taiwan in (*c*) 2005; (*d*) 2008.

**Fig. 4 (a, b).**
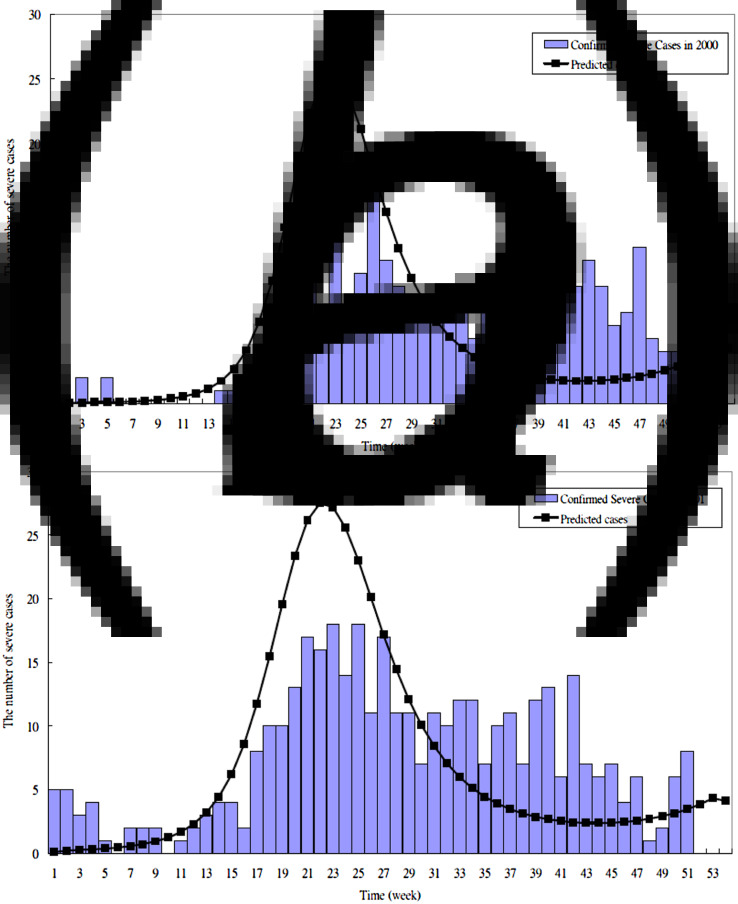
The observed and predicted severe HFMD cases in Taiwan in (*a*) 2000; (*b*) 2001.

**Fig. 4 (c, d).**
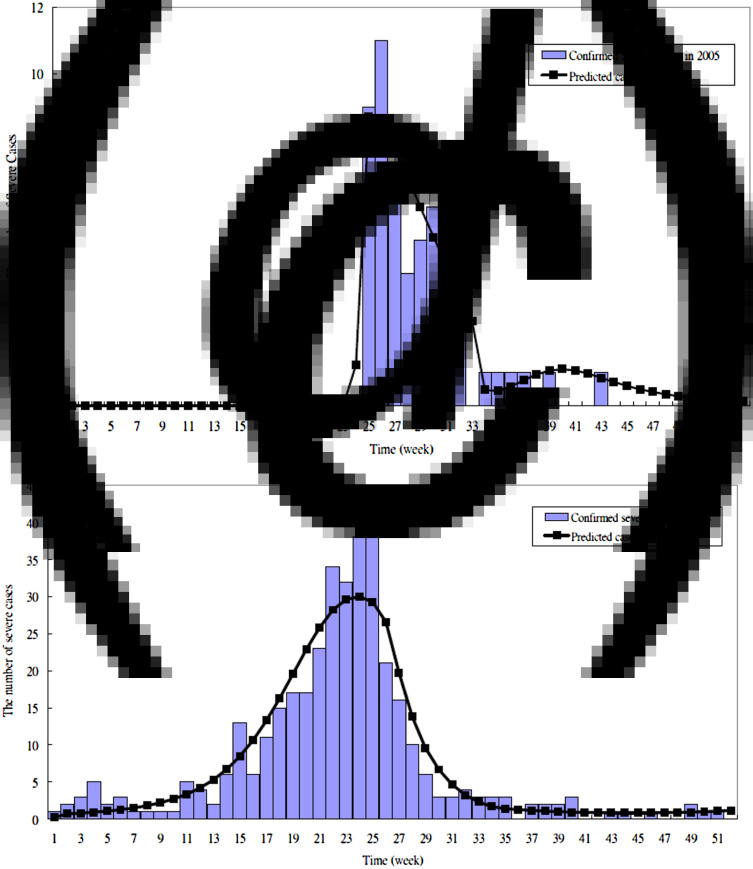
The observed and predicted severe HFMD cases in Taiwan in (*c*) 2005; (*d*) 2008.

**Table 2.** The results of associated parameters for model fitting the outbreaks

Variable	Range	Outbreak 2000	Outbreak 2001	Outbreak 2005	Outbreak 2008	Outbreak 2008*
*β* (per day)	—	5·7 × 10^−7^	5·6 × 10^−7^	4·5 × 10^−7^ (<34 weeks) 1·2 × 10^−6^ (⩾34 weeks)	5·3 × 10^−7^ (⩽25 weeks) 2 × 10^−7^ (25–33 weeks) 4 × 10^−7^ (⩾34 weeks)	Inverse gamma (2·00, 5·85 × 10^−7^)
*δ* (per day)	0–0·21	0·0018	0·0023	0 (<24 weeks) 6·75 × 10^−4^ (24–32 weeks) 1·35 × 10^−4^ (>32 weeks)	0·0036	0·0036
Proportion of cases reported	—	25%	22%	22·5%	22%	22%
Predicted severe cases	—	365·38	403·7	66·63	373·52	
(reported cases)		(367)	(395)	(57)	(373)	—
*R*_0_	—	1·22	1·21	1·59	1·18	1·37
						(95% CI 0·23–5·71)

*δ*, Proportion of severe case; *β*, transmission coefficient; CI, confidence interval.

* The results in sensitivity analysis after 15 000 times sampling simulation.

**Fig. 5.**
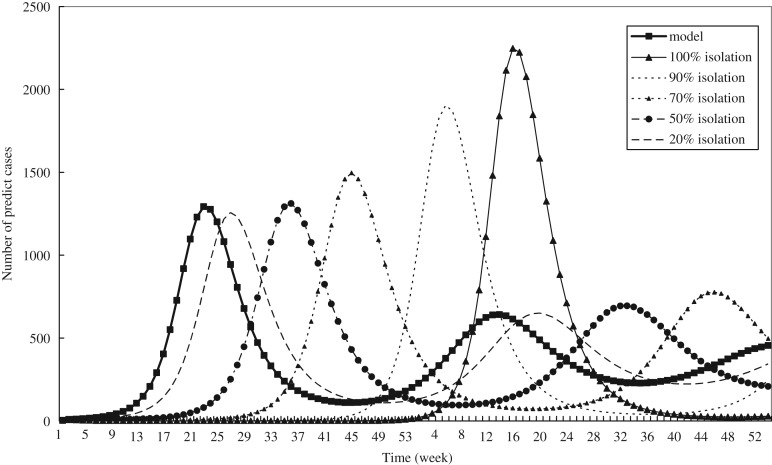
The results of an isolation strategy for HFMD at different isolation rates.

The results of the simulations on the delayed time and total number of HFMD cases with different proportions of isolation strategy (Ω), assuming that 0·2, 0·5, 0·7, 0·9 or 1 were applied, are shown in Figure 5. The delayed time for generating an epidemic curve varying with the proportion of isolation ranged from 4 weeks for 20% isolation, 13 weeks for 50%, 22 weeks for 70%, 37 weeks for 90%, and 47 weeks for 100% isolation. After 4 years of continuing the same strategy, the total number of HFMD cases fell by 1·3% for 20% isolation, 4·7% for 50%, 6·6% for 70%, 13·0% for 90%, and 13·3% for 100% isolation compared to no isolation strategy.

### Computation of *R*_0_ with the Bayesian MCMC method

We computed the basic reproductive number (*R*_0_) by deriving the posterior distribution of *R*_0_, taking into account the variability of *R*_0_ that results from the uncertainty of the parameters incorporated into the dynamic modified SIR model as mentioned above. The proper statistical distributions of these parameters (*β, α, τ*_a,_ *τ*_s_, *ρ*), according to the findings from these outbreaks in 2000, 2001, 2005 and 2008 (Supplementary material), were assigned as stated in the Methods section. After 5000 burn-in samples, we ran another 10 000 samples based on five parameters (*β, α, τ*_a,_ *τ*_s_, *ρ*) given a fixed death rate (*μ*). The *R*_0_ of HFMD was estimated as 1·37 [95% confidence interval (CI) 0·24–5·84] based on the data from 2008. The convergence was plotted as shown in Supplementary Figure S1. Note that the convergence was achieved after 10 000 samples (including 5000 burn-in samples). To guarantee the convergence, we ran an extra 5000 iterations, and the results were very similar.

## DISCUSSION

We built a compartmental SIR model by incorporating information on the surveillance data to estimate the *R*_0_ (1·37, 95% CI 0·24–5·84) of HFMD using data from a series of outbreaks in different calendar years. Previous studies on the estimation of *R*_0_ were limited. The isolation strategy against HFMD was not only able to delay the onset of the outbreak but also to decrease the disease burden of HFMD, i.e. the total number of HFMD cases.

Ma *et al*. reported that the median *R*_0_ of EV71 and CVA16 were 5·48 and 2·5, respectively, in Hong Kong [17]. The assumption of only one index case at the initial phase and the neglect of household transmission resulted in the overestimation of the reproductive numbers in that study. In addition, our estimated *R*_0_ concurrently denoted the combined effects of HFMD and these viruses. We simulated the occurrence of HFMD throughout the entire year, and we also allowed the initial number of cases to change, not only one index case. Hence, the *R*_0_ of HFMD estimated in our study was therefore lower than the value in the Ma *et al*. study.

One of the unique characteristics of our study was the prediction of HFMD outbreak by different calendar years. The values of beta used were 4·5 × 10^−7^ (<34 weeks) and 1·2 × 10^−6^ (⩾34 weeks) for the two periods, but the other parameters were not changed. The two peaks of the epidemic curve could be simulated in the 2005 outbreak. According to the laboratory surveillance data, EV71 was the major cause of these outbreaks. In 2005, the major cause of the first wave was EV71, and the second wave was due to CVA16. Therefore, the different ratios of severe cases to all symptomatic cases (*δ*) were noted. Similarly, the different transmission rates (*β*) in different periods were also found. Hence, we obtained an asymmetric epidemic curve such as that in Figure 3*d* for the 2008 outbreak. However, the major cause was still only EV71. This is supported by the findings that the higher ratio of severe cases (*δ*) is related to the higher activity of EV71.

We observed a poor fit of the predicted HFMD cases in both 2000 and 2001 (Supplementary Table S1). There are two explanations for this. In the early stage, the sentinel surveillance system for HFMD may be unstable because clinical physicians were unfamiliar with the reporting system or the characteristics of severe cases of HFMD. Therefore, the proportion of severe HFMD cases fluctuated greatly in the early outbreak. The second reason is that HFMD has different aetiological viruses in different seasons and years, resulting in a wide range of prevalence figures. Both aspects account for the poor fit of the predicted severe HFMD cases in both 2000 and 2001 (Supplementary Table S1).

The proportion of severe cases (*δ*) was different in these outbreaks because of the different proportion of the aetiologies. The major aetiologies were EV71 and CVA16 in 2000, 2001 and 2008. The first wave of the outbreak resulted from viruses including CVA16 and EV71 in 2005. The second wave of the outbreak resulted from viruses including CVB3 and EV71 in 2005. From the epidemic curve of severe cases with enterovirus, we believe that EV71 was more active in the first outbreak wave because the cases with EV71 had a high mortality rate, which was captured by a higher value of *δ*.

### Implications for the control of HFMD

Our proposed model is based not only on the dynamic infectious status but also on dynamic changes in the disease with complications. Incorporating the information about the clinical symptoms or disease complications that were commonly seen in routine surveillance data into the proposed model would make the model as accurate as possible in order to emulate the real scenario on infection as well as disease process. This is useful for forestalling the spread of HFMD according to *R*_0_ and predicting the expected sequelae of subsequent outbreaks on the basis of information available from early epidemic curves with the same pathogen of virus variants. Providing such information may be of great benefit to evaluate the effectiveness of certain preventive strategies such as a health isolation strategy for policy-makers as shown in the current study.

By the application of these parameters and *R*_0_, we demonstrated that the isolation strategy against HFMD delayed the epidemic peak and also reduced the burden of HFMD. The proposed model is also very flexible in evaluating further aggressive preventive action such as quarantine before the development of vaccine. Moreover, our proposed dynamic model provides a good foundation for the future effectiveness and cost-effectiveness analysis of a vaccine against HFMD as the implementation of a vaccine against HFMD (EV71, CVA16) is the best policy for preventing severe enterovirus cases.

There were unavoidable biases in estimating the *R*_0_ of HFMD because it was limited to the assumptions of the model, such as a homogeneous random mixing population, the fixed ratio of the number of reported cases in sentinel surveillance systems to the number of all cases, and the estimated parameters derived only from the characteristics of CVA16 and EV71. To minimize these biases, we estimated the parameters based on the comprehensive empirical data from infection until the occurrence of clinical complications. More importantly, the credibility of our model is attributed to the fact that there were reliable severe cases with enterovirus reported in the system, which enabled us to estimate the ratio of reported cases in the surveillance system to all cases in Taiwan and to further stabilize the estimation of relevant parameters contained in the model. Furthermore, HFMD has different aetiological causes, and the prevalence of the different viruses is different in different seasons, but we assumed that all of their characteristics were the same as those of EV71 or non-EV71 (CVA16). Another limitation is that the parameters of the model were only estimated from the characteristics of CVA16 and EV71 because we were more concerned with the impact of the severe cases of enterovirus, with their major aetiologies being composed of CVA16 and EV71. In addition, HFMD can be transmitted by asymptomatic persons. However, no studies reported on the difference of transmission probability between symptomatic and asymptomatic persons. For the ease of computation, we assumed that there were no differences between them.

Partly because we are interested in large outbreaks of severe cases of HFMD, and partly because the characteristics of severe cases of HFMD outbreaks depended on the enteroviruses (EV71 or CVA16) in different years, we applied the dynamic epidemic model to fit the surveillance data in each year and not all years. However, bias may be present in the analysis of isolation strategy (Ω) because we did not take seasonal variation into account.

In conclusion, we show how to estimate *R*_0_ in the light of the parameters of the proposed dynamic model with accuracy by incorporating the information of Taiwanese sentinel surveillance data and clinical symptoms or disease complications. The estimated *R*_0_ was 1·37, suggesting a higher likelihood of the spread of HFMD if no infection control policy is envisaged. Based on our simulation results, we also demonstrated that the isolation strategy against HFMD not only delayed the spread of HFMD with a range from 4 weeks for 20% isolation to 47 weeks for 100% isolation but also reduced total number of HFMD cases with a range from 1·3% reduction for 20% isolation to 13·3% reduction for 100% isolation. The developed model can also be easily used to evaluate the effectiveness of other control strategies including quarantine and vaccination (if the vaccine can be developed) in order to reduce the chance of the spread of HFMD.

## Supplementary material

For supplementary material accompanying this paper visit https://doi.org/10.1017/S0950268815002630. click here to view supplementary material

## References

[1] Melnick JL. Enteroviruses: Polioviruses, Coxsackieviruses, Echoviruses, and Newer Enteroviruses 3rd edn. Philadelphia: Lippincott-Raven Publishers, 1996, pp. 655–712.

[2] Lum LC, Echovirus 7 associated encephalomyelitis. Journal of Clinical Virology 2002; 23: 153–160. 11595594 10.1016/s1386-6532(01)00214-1

[3] Apisarnthanarak A, Echovirus type 11: outbreak of hand-foot-and-mouth disease in a Thai hospital nursery. Clinical Infectious Diseases 2005; 41: 1361–1362. 16206118 10.1086/497076

[4] Kushner D, Caldwell BD. Hand-foot-and-mouth disease. Journal of the American Podiatric Medical Association 1996; 86: 257–259. 8699347 10.7547/87507315-86-6-257

[5] Schmidt NJ, Lennette EH, Ho HH. An apparently new enterovirus isolated from patients with disease of the central nervous system. Journal of Infectious Diseases 1974; 129: 304–309. 4361245 10.1093/infdis/129.3.304

[6] Ho M. Enterovirus 71: the virus, its infections and outbreaks. Journal of Microbiology, Immunology and Infection 2000; 33: 205–216. 11269363

[7] Chang LY, Risk factors of enterovirus 71 infection and associated hand, foot, and mouth disease/herpangina in children during an epidemic in Taiwan. Pediatrics 2002; 109: e88. 12042582 10.1542/peds.109.6.e88

[8] Chang LY, Comparison of enterovirus 71 and coxsackievirus A16 clinical illnesses during the Taiwan enterovirus epidemic, 1998. Pediatric Infectious Disease Journal 1999; 18: 1092–1096. 10608631 10.1097/00006454-199912000-00013

[9] Monto Ho MD, An epidemic of enterovirus 71 infection in Taiwan. New England Journal of Medicine 1999; 341: 929–935. 10498487 10.1056/NEJM199909233411301

[10] Diekmann O, Heesterbeek J. Mathematical Epidemiology of Infectious Diseases: Model Building, Analysis and Interpretation. New York: Wiley, 2000.

[11] Lin TY, Enterovirus 71 outbreaks, Taiwan: occurrence and recognition. Emerging Infectious Disease 2003; 9: 291–293. 10.3201/eid0903.020285 PMC2963902 12643822

[12] R.O.C. (Taiwan) DoH. Taiwan Public Health Reports. Taiwan; 2000, 2001, 2005, 2008.

[13] Chang L-Y, Transmission and clinical features of enterovirus 71 infections in household contacts in Taiwan. Journal of the American Medical Association 2004; 291: 222–227. 14722149 10.1001/jama.291.2.222

[14] Goh KT, An outbreak of hand, foot, and mouth disease in Singapore. Bulletin of the World Health Organization 1982; 60: 965–969. 6297819 PMC2535982

[15] Heymann DL. Control of Communicable Disease Manual, 18th edn. American Public Health Association, 2004.

[16] Liu CC, An outbreak of enterovirus 71 infection in Taiwan, 1998: epidemiologic and clinical manifestations. Journal of Clinical Virology 2000; 17: 23–30. 10814935 10.1016/s1386-6532(00)00068-8

[17] Ma E, Estimation of the basic reproduction number of enterovirus 71 and coxsackievirus A16 in hand, foot, and mouth disease outbreaks. Pediatric Infectious Disease Journal 2011; 30: 675–679. 21326133 10.1097/INF.0b013e3182116e95

