# Supplementary material for: A dynamic model for the outbreaks of hand, foot, and mouth disease in Taiwan
Source: Epidemiol Infect. 2015 Nov 16;144(7):1500–11. doi: 10.1017/S0950268815002630 (PMC4823833; doi:10.1017/S0950268815002630)
Supplement: Supplementary file 1 [file S0950268815002630sup001.doc]

**SUPPLEMENTARY MATERIAL**

**Methods**

***The HFMD Model***

We modified the SIR model with clinical symptoms or disease complications. It is divided into nine compartments. Individuals moved from the susceptible (S) to infectious cases before developing into disease (I) with the force of infection at time t [λ(t)]. The nonlinear ordinary differential equations are as follows.

$\frac{dS}{dt}=\mu_{1}N-(\lambda(t)+\mu)S(t)$ (1)

μ_1_: the birth rate; μ: the death rate; N: the number of total population; S(t): the number of susceptible population at time t.

The force of infection is proportional to the β×[I(t)＋ASI(t)＋(1-Ω)EVI(t)], where β denotes the transmission coefficient, I(t) denotes the number of infectious cases before developing into disease at time t, ASI(t) denotes the number of asymptomatic disease with infectiousness (AS-I) at time t, EVI(t) denotes the number of infectious cases after showing symptoms at time t.

In order to assess the impact of isolation strategy, Ω is denoted to represent the ratio of isolated EV-I. Infectious cases (I) developed to asymptomatic disease (AS-I) or symptomatic disease (EV-I) at the transmission rate α. The nonlinear ordinary differential equations are formulated as follows.

$\frac{dI}{dt}=\lambda S-(\alpha+\mu)I(t)$ (2)

The recovery rate of AS-I subjects was $\tau_{a}$. The rate of developing the symptomatic cases with immunity (EV-R) for EV-I subjects is denoted as $\tau_{s}$. The differential equations are as follows.

$\frac{dASI}{dt}=\alpha(1-\rho)I(t)-(\mu+\tau_{a})ASI(t)$ (3)

$\frac{dEVI}{dt}=\alpha\rho I(t)-(\mu+\tau_{s})EVI(t)$ (4)

Here ρ denotes the ratio of symptomatic cases. The symptomatic cases with immunity (EV-R) developed to typical cases with hand foot and mouth disease (HFMD), severe cases (C) and other mild symptoms or herpangina (HA) at transmission rate γ. The differential equations are as follows.

$\frac{dEV\text{R}}{dt}=\tau_{s}EVI(t)-(\mu+\gamma)EVR(t)$ (5)

Therefore, the dynamic change of R, HA, HFMD and C per unit time are as follows.

$\frac{dR}{dt}=\tau_{a}ASI(t)-\mu R(t)$ (6)

$\frac{dHA}{dt}=\gamma(1-\delta-\theta)EV\text{R}(t)-\mu HA(t)$ (7)

$\frac{dHFMD}{dt}=\gamma\theta EVR(t)-\mu HFMD(t)$ (8)

$\frac{dC}{dt}=\gamma\delta EVR(t)$ (9)

γ: the transfer rate of observation period; θ: the ratio of the HFMD; δ: the proportion of severe cases to all symptomatic HFMD cases.

All severe case of HFMD would be presumably reported and the ratio of the number of cases with HFMD reported in sentinel Surveillance Systems to the number of all cases with HFMD is fixed at any time. Waning immunity was also neglected. The model was built under the assumption of homogenous random mixing population. All outbreaks begun from the infected cases reported in sentinel Surveillance Systems before the occurrence of outbreak. We assumed all susceptible could be infected with one of these viruses among the same outbreak within one year. After one of these enterovirus infections, the persons obtained the immunity and could be protected against the attack of other enterovirus in short period.

***Derivation of basic reproductive number (R_0_) by next generation method***

By referring to next generation method [10] that is a general method of deriving R_0_ in models with several disjoint compartments. R_0_ is defined as

$\text{R}_{0}=\rho\text{(FV}^{\text{-1}}\text{)}$ (10),

where $\rho\text{( }\cdot\text{ )}$ is the matrix dominant eigenvalue (spectral radius), F is the newly infection rate matrix, and V is the transition matrix between compartments.

In our HFMD model, there are more compartments defined for infectious states defined by disease status, including I, AS-I, and EV-I. We assume the number of I can be produced by I, AS-I, or EV-I at the same rate of$\beta$. Then, the F matrix and V matrix can be written on the basis of the equations 1-9 shown in Appendix.

$\begin{matrix} \begin{matrix} \text{ I} & \text{ ASI} & \text{EVI} \end{matrix} \\ \text{F}=\begin{matrix} \text{I} \\ \text{ASI} \\ \text{EVI} \end{matrix}\left(\begin{matrix} \beta& \text{ }\beta& \text{ }\beta\\ 0 & \text{ 0} & \text{ 0} \\ 0 & \text{ 0} & \text{ 0} \end{matrix}\right) \end{matrix}$ and

$\begin{matrix} \begin{matrix} \text{ I} & \text{ ASI} & \text{ EVI} \end{matrix} \\ \text{V}=\begin{matrix} \text{I} \\ \text{ASI} \\ \text{EVI} \end{matrix}\left(\begin{matrix} \mu+\alpha& 0 & 0 \\ \text{-}\alpha\text{(1-}\rho\text{)} & \mu+\tau_{\text{a}} & 0 \\ \text{-}\alpha\rho& 0 & \mu+\tau_{\text{s}} \end{matrix}\right) \end{matrix}$ .(11)

Hence, the basic reproductive number derived from our model is given by the formula:

$\text{R}_{0}=\frac{\beta}{\mu+\alpha}+\frac{\beta\alpha\text{(1-}\rho\text{)}}{\text{(}\mu+\alpha\text{)(}\mu+\tau_{\text{a}}\text{)}}+\frac{\beta\alpha\rho}{\text{(}\mu+\alpha\text{)(}\mu+\tau_{\text{s}}\text{)}}$, (12)

where β :the transmission rate; μ: denotes the death rate; α: the transition rate from I (infectious cases before developing symptoms) to EV-I (infectious cases after developing symptoms) or AS-I (asymptomatic cases with infectiousness); ρ: the proportion of symptomatic cases; τ_a_: the rate of recovery from AS-I; τ_s_: the rate of recovery from EV-I.

***Goodness of fit***

The results of observed and predicted values for the goodness of fit are listed in Table S1. However, the statistic was chi-square =383.23 with 33 degrees of freedom (p<0.0001) for critical cases in 2000, and chi-square =747.51 with 39 degrees of freedom ((p<0.0001) for critical cases in 2001. If we want to get the better simulated values, the value of δ will be highly fluctuated in the same period of outbreaks. It seemed not reasonable because the proportion of severe cases with HFMD should be alike in the same virus. Therefore, we chose the parameters to fit the total number of severe cases and epidemic peak. The statistic was chi-square =0.6888 with one degrees of freedom (p=0.4066) for severe cases in 2000, and chi-square =0.3739 with one degrees of freedom ((p=0.5409) for severe cases in 2001.

**Calculating R_0_ by MCMC**

We estimated the parameters of HFMD model according to the findings from these outbreaks in 2000, 2001, 2005 and 2008. From the next generation method, we know that β, α, τ_a_, τ_s_, and ρ are the determinant parameters. So we defined these parameters except for μ (death rate) as proper distribution for sensitivity analysis.

We defined β ~ inverse gamma distribution (k,q), so the mean of β was q/(k-1) and the variance of β was q^2^/[(k-1) ^2^* (k-2)]. Because the mean of β was estimated as 5.85*10^-7^and the variance was estimated as 10.971*10^-7^ from the findings of outbreaks, we can easy to find that k was equal to 2.000, and q was 5.85*10^-7^. We defined α as gamma distribution (a, b) and the mean of α was 0.35 from the findings of outbreaks. We assumed that the distance between lower bound (0.167) and mean (0.35) was 3 times of standard deviation. Therefore, the variance can be estimated as 0.003721. Because a*b=0.35 and a*b^2^= 0.003721, we can get the a is 32.92 an b is 0.01. We defined τ_a_ and τ_s_ as gamma distributions and the means were 0.08 from the findings of outbreaks. We assumed that the distance between upper bound (0.125) and mean (0.08) was 3 times of standard deviation. Therefore, the variance can be estimated as 0.000225. Likewise, we can get the τ_a_, τ_s_ ~ gamma(28.44, 0.0028125). We defined ρ as beta distributions (c, d) and the means were 0.7 from the findings of outbreaks. We assumed that the distance between upper bound (0.94) and mean (0.7) was 3 times of standard deviation. So, the variance can be estimated as 0.0064. Because the mean c/(c+b) = 0.7 and the variance c*d/[(c+d+1)*(c+d)^2^] = 0.0064, we can get the c is 0.6225 and d is 0.26048.

We defined these parameters (α, τ_a_, τ_s_, ρ, β) to be as the above distribution and applied the other parameters from outbreak in 2008. After 15000 times of sampling simulation with burn-in interval of 5000 times and thinning interval of one, the mean of R_0_ was estimated as 1.37 (95% CI: 0.24-5.84) by MCMC.

Table S1. Results of observed and predicted values for goodness of fit

|  | Outbreak in 2000 | |  | Outbreak in 2001 | |
| --- | --- | --- | --- | --- | --- |
| Week | Observed values | Predicted values | Week | Observed values | Predicted values |
| 3-14 | 5 | 5.4 | 1-12 | 35 | 9.0 |
| 15-17 | 5 | 13.0 | 13 | 5 | 3.2 |
| 18-19 | 7 | 23.0 | 14-15 | 9 | 10.6 |
| 20 | 8 | 18.2 | 16-17 | 14 | 20.3 |
| 21 | 15 | 22.3 | 18 | 12 | 15.5 |
| 22 | 7 | 24.9 | 19 | 19 | 19.5 |
| 23 | 13 | 25.3 | 20 | 16 | 23.3 |
| 24 | 7 | 23.9 | 21 | 15 | 26.2 |
| 25 | 10 | 21.1 | 22 | 14 | 27.5 |
| 26 | 17 | 17.9 | 23 | 17 | 27.2 |
| 27 | 11 | 14.8 | 24 | 14 | 25.6 |
| 28 | 9 | 12.0 | 25 | 27 | 23.0 |
| 29 | 7 | 9.6 | 26 | 11 | 20.1 |
| 30 | 7 | 7.8 | 27 | 27 | 17.1 |
| 31 | 8 | 6.3 | 28 | 17 | 14.4 |
| 32 | 8 | 5.2 | 29 | 16 | 12.1 |
| 33 | 9 | 4.3 | 30 | 7 | 10.1 |
| 34 | 5 | 3.6 | 31 | 14 | 8.4 |
| 35 | 7 | 3.1 | 32-33 | 13 | 13.1 |
| 36-37 | 13 | 5.0 | 34-35 | 8 | 9.6 |
| 38 | 5 | 2.1 | 36 | 13 | 3.9 |
| 39-40 | 12 | 3.8 | 37 | 12 | 3.5 |
| 41 | 14 | 1.8 | 38-39 | 10 | 6.0 |
| 42 | 9 | 1.7 | 40-41 | 7 | 5.2 |
| 43 | 11 | 1.7 | 42 | 9 | 2.4 |
| 44 | 9 | 1.8 | 43 | 7 | 2.4 |
| 45 | 6 | 1.8 | 44-45 | 5 | 4.8 |
| 46 | 7 | 1.9 | 46-47 | 7 | 5.0 |
| 47 | 12 | 2.1 | 48-50 | 8 | 8.7 |
| 48 | 5 | 2.3 | 51 | 7 | 3.5 |
| 49-50 | 8 | 5.4 | - | - | - |
| 51-53 | 14 | 11.4 | - | - | - |
| Total | 290 | 304.5 |  | 395 | 381.1 |
| χ^2^ |  | 380.66 |  |  | 219.9 |
| p value |  | <0.0001 |  |  | <0.0001 |

Table S1 Results of observed and predicted values for goodness of fit (*cont.*)

|  | Outbreak in 2005 | | |  | Outbreak in 2008 | |
| --- | --- | --- | --- | --- | --- | --- |
| Week | Observed values | Grouping Observed values | Predicted values | Week | Observed values | Predicted values |
| 24 | 0 | 0 | 1.2431 | 1-10 | 20 | 13.3051 |
| 25 | 9 | 9 | 8.6963 | 11 | 5 | 3.3718 |
| 26 | 11 | 11 | 8.3876 | 12-13 | 6 | 9.5481 |
| 27 | 7 | 7 | 7.7423 | 14 | 6 | 6.7304 |
| 28 | 4 |  |  | 15 | 13 | 8.5064 |
| 29 | 5 | 9 | 12.8816 | 16 | 6 | 10.6907 |
| 30 | 6 | 6 | 5.0825 | 17 | 17 | 24.0039 |
| 31 | 5 |  |  | 18 | 15 | 16.3246 |
| 32 | 4 | 9 | 7.7969 | 19 | 17 | 19.5891 |
| 33 | 0 |  |  | 20 | 17 | 22.873 |
| 34 | 1 |  |  | 21 | 23 | 25.8537 |
| 35 | 1 |  |  | 22 | 34 | 28.2018 |
| 36 | 1 |  |  | 23 | 32 | 29.61 |
| 37 | 1 |  |  | 24 | 38 | 29.979 |
| 38 | 0 |  |  | 25 | 39 | 29.2563 |
| 39 | 1 |  |  | 26 | 21 | 26.5615 |
| 40 | 0 |  |  | 27 | 16 | 19.7453 |
| 41 | 0 |  |  | 28 | 10 | 13.7927 |
| 42 | 0 |  |  | 29 | 6 | 9.5835 |
| 43 | 1 | 6 | 10.8649 | 31 | 6 | 11.3359 |
|  |  |  |  | 33 | 7 | 5.5765 |
|  |  |  |  | 35 | 6 | 3.1081 |
|  |  |  |  | 36-39 | 6 | 4.517 |
|  |  |  |  | 40-44 | 5 | 4.4127 |
|  |  |  |  | 45-51 | 5 | 6.5475 |
| Total | 57 |  |  |  | 370 | 383.025 |
| χ^2^ |  |  | 5.84 |  |  | 31.83 |
| p value |  |  | 0.6651 |  |  | 0.1630 |

Figure S1.
